# *Plesiomonas shigelloides* as an Emerging Pathogen in Catfish Aquaculture: A Case from a South Texas Commercial Farm

**DOI:** 10.3390/microorganisms14010144

**Published:** 2026-01-08

**Authors:** Haitham H. Mohammed, Noha I. ElBanna, Ozgur Erdogan, Suja Aarattuthodi, Hasan C. Tekedar, Hossam Abdelhamed, Josué Díaz-Delgado

**Affiliations:** 1 Department of Rangeland, Wildlife and Fisheries Management, Texas A&M University, College Station, TX 77843, USA; noha.elbana.fish@suez.edu.eg (N.I.E.);; 2 Plant Genetics Research Unit, United States Department of Agriculture—Agricultural Research Service, Columbia, MO 65211, USA; 3 College of Veterinary Medicine, Mississippi State University, Starkville, MS 39762, USA; 4 Texas A&M Veterinary Medical Diagnostic Laboratory, College Station, TX 77843, USA

**Keywords:** fish, opportunistic pathogen, infection, disease, gastroenteritis, outbreak, mortality, food safety, multidrug-resistance

## Abstract

During the summer of 2023, a spontaneous disease outbreak occurred in intensively stocked hybrid catfish (♀ channel catfish, *Ictalurus punctatus* × ♂ blue catfish, *I. furcatus*) in earthen ponds on a commercial aquaculture farm in South Texas. The farmer reported 50 to 80 dead fish per pond daily for a month. The fish were market size (1.0 ± 0.3 kg on average), resulting in substantial economic losses. Fifteen moribund fish were submitted for laboratory examination. Grossly, the fish showed distended abdomens, erythematous fins, and inflamed vents. Autopsy demonstrated visceral congestion, distended gastrointestinal tracts, and serosanguineous peritoneal effusion. Bacterial cultures from the internal organs revealed homogeneous bacterial growth after incubation. Presumptive biochemical characterization of the isolated bacteria identified *Plesiomonas shigelloides*. Further molecular confirmation was achieved by species-specific PCR amplification and 16S-rRNA sequencing. Juvenile catfish were experimentally challenged with the recovered isolates to fulfill Koch’s postulates. Moreover, an antibiogram was performed to evaluate the susceptibility of the isolates to a panel of FDA-approved antimicrobials. *P. shigelloides* isolates were pathogenic to channel catfish and alarmingly multidrug-resistant. We report here, for the first time, *P. shigelloides* infection in Texas commercial catfish aquaculture, emphasizing its significance as an emerging enteric pathogen that is difficult to treat with FDA-approved antimicrobials.

## 1. Introduction

Catfish farming represents the largest sector of U.S. aquaculture, contributing ~70% of the total finfish aquaculture production and playing a vital role in the domestic economy [1,2]. In 2024, the U.S. catfish industry generated $358 million in sales, as reported by the USDA National Agriculture Statistics Service (USDA-NASS) [3]. The top four catfish-producing states—Mississippi, Alabama, Arkansas, and Texas—accounted for 95% of the nation’s total catfish sales, with most of the catfish (~95%) being raised in earthen ponds. In Texas alone, commercial catfish production spans ~1600 water surface acres [3]. Despite its economic significance, the U.S. catfish industry has long been constrained by disease problems, which remain a primary challenge to its growth and profitability [4].

As with other intensive agricultural systems, catfish farming suffers from a variety of disease problems resulting from intensive production practices and suboptimal culture conditions. These include infectious agents (such as bacterial agents, viruses, parasites, and fungal or oomycete pathogens), as well as non-infectious stressors (e.g., environmental hypoxia, temperature fluctuations/extremes, poor water quality, nutritional deficiencies, handling and transport stress, and toxic algal blooms). These factors can act independently or synergistically to impair fish health and often predispose fish to secondary infections. Among these, infectious diseases pose the greatest threat to catfish farming, with bacterial pathogens being the leading cause of economic losses. Bacterial diseases account for more disease outbreaks in catfish aquaculture than all other causes combined [5,6,7,8]. Recent epidemiological data from the Alabama Fish Farming Center highlights the severity of this issue, reporting that bacterial infections were responsible for more than 83% of all catfish disease-related mortalities, over five times the losses attributed to non-bacterial causes [9].

*Plesiomonas shigelloides* (formerly known as *Aeromonas shigelloides*) is a Gram-negative, weakly motile, facultative anaerobic, rod-shaped bacterium and the only species representing the genus *Plesiomonas*. The genus *Plesiomonas* was under the family Vibrionaceae for many years but has been reclassified and currently resides in the family Enterobacteriaceae; it is the only oxidase-positive member in this family [10]. It inhabits diverse environments and is widely distributed in aquatic ecosystems. This bacterium has been isolated from streams, rivers, lakes, estuarine waters, amphibians, reptiles, crustaceans, fish, birds, and mammals, including humans [11,12]. There is an abundance of *P. shigelloides* in catfish gut microbiota, pond sediment, and pond water [13,14]. In humans, usually, *P. shigelloides* is an opportunistic enteric pathogen causing bacterial gastroenteritis, diarrhea, and in some cases extraintestinal infections, such as wound infection, bacteremia, meningitis, and pseudoappendicitis [15,16,17,18,19,20]. Additionally, *P. shigelloides* has been recognized as a foodborne and waterborne pathogen, causing disease associated with consumption of contaminated water, raw or undercooked fish, crustaceans, and shellfish [21,22].

A natural disease outbreak occurred in several ponds on a commercial catfish farm in South Texas during the hot summer months of 2023, resulting in significant fish mortality and economic losses. Mortalities began in late June and peaked in early July 2023. The farmer contacted the Fish Health & Disease Laboratory (FHDL) at Texas A&M University and submitted samples for disease diagnostics. This study aimed at examining the causative agent responsible for this epizootic disease and exploring a possible treatment/control strategy. We report here, for the first time, the isolation, characterization, and pathogenicity of multidrug-resistant *P. shigelloides* isolates from commercially farmed catfish in Texas aquaculture.

## 2. Materials and Methods

### 2.1. Epizootic Description and Fish Sample Collection

On a commercial catfish farm in South Texas, a disease outbreak caused by a natural infection resulted in daily fish mortalities for four consecutive weeks in an open-earthen pond system during the summer of 2023. The farm consists of 60 rectangular-shaped ponds of varying sizes (1–8 acres) with an average depth of 1.6 ± 0.2 m. The production ponds are supplied with aerated well water with no water exchange and have been in production for a decade. All the ponds were intensively stocked with hybrid catfish (♀ Channel catfish *Ictalurus punctatus* × ♂ Blue catfish *I. furcatus*), originally sourced from a catfish hatchery in Sunflower, Mississippi. The stocking size and biomass of fish per pond varied according to the production stage, but were generally stocked with 20,000–25,000 lbs per acre.

The mortality was observed in twenty-two growout ponds, approximately one year after stocking with 7-month-old, 8–10 cm-long catfish fingerlings. The ponds with fish succumbing to the disease had larger catfish, ~1.0 ± 0.3 kg in average size, while the rest of the unaffected ponds were stocked with smaller hybrid catfish (0.4–0.6 kg average). The larger fish were fed once daily to satiation with 32% protein floating catfish pellets manufactured by a commercial feed mill (Rangen, Inc., Buhl, ID, USA), reflecting a high feeding rate, while the smaller fish received pellets (same feed) at 2% of their estimated total body weight per day. The mortalities started in late June, and peaked in the first week of July, corresponding with a period of highest water temperature, lowest dissolved oxygen (DO), and highest feeding rates. Water quality parameters were monitored routinely by farm personnel as part of standard commercial production practices and are maintained within adequate limits for catfish production. Summary data (parameter averages and ranges) were provided to FHDL as part of the diagnostic case history. During the period of mortality, total ammonia nitrogen was 1.91 ± 0.20, and pH was 8.11 ± 0.04. The water temperature in the affected ponds ranged from 28.7 °C to 32.2 °C. The farm uses a controlled intensive mechanical aeration system programmed to activate when DO concentrations fall below 3.0 ppm to maintain DO above this threshold. DO levels during the mortality period varied daily and ranged from 2.9 ppm to 7.3 ppm, with the minimum value representing a transient measurement prior to aerator activation.

Moribund hybrid catfish (n = 15) displaying signs of infection were collected using hand and cast nets, placed individually in sterile bags, and transported chilled on ice (approximately 4 °C) in insulated coolers to the FHDL at Texas A&M University for disease diagnosis.

### 2.2. Autopsy and Bacterial Isolation

Routine diagnostic procedures were performed upon arrival at the FHDL, including external examination, microbiological testing, and molecular analysis following standard laboratory protocols [23]. Clinical signs and gross lesions were noted and recorded. Fresh preparations, including skin and mucus scrapings, fin biopsies, and gill biopsies were examined microscopically for the presence of external pathogens (2 replicate wet mounts per fish). An Olympus SZ6145 binocular stereo microscope and an Olympus BX53 Phase with DP23 Camera upright microscope (Olympus, Tokyo, Japan) were used to view all preparations. Fish were then surface sterilized with 75% alcohol before opening their coelomic cavities. Bacterial isolation was aseptically performed from the main internal organs (liver, spleen, posterior kidney, brain, and ascitic fluid) on 5% sheep blood agar (Hardy Diagnostics, Santa Maria, CA, USA) and Trypticase Soy Agar (TSA; Becton Dickinson, Sparks, MD, USA) plates. Individual colonies were picked and subcultured under the same conditions for purification after 48 h incubation at 28 °C.

The internal examination also included microscopic examination of tissue squash preps of the liver, kidneys, and spleen. Sampling for the presence of viruses, using the channel catfish ovary (CCO, source ATCC) cell line obtained from cell repositories of the Texas Parks and Wildlife Department State Fish Hatchery, followed the methods outlined in the USFWS and AFS-FHS Blue Book 2020 [24]. The CCO cell line is the cell line of choice for isolation of channel catfish virus (CCV), the most significant viral pathogen affecting catfish aquaculture in the United States. Briefly, kidney and spleen tissue samples were collected, placed in Hank’s Balanced Salt Solution, homogenized, filtered, diluted (1:1), and inoculated onto CCO cells. The samples were incubated at 25 °C for 10 days and were monitored for the development of cytopathic effect (CPE). After the initial incubation period, a blind passage was performed by transferring a portion of the supernatant to a fresh culture flask with a healthy CCO cell monolayer to detect low-titer viruses. No histopathological examination of tissue samples from the submitted fish was performed.

### 2.3. Bacterial Identification

Pure cultures of selected representative bacterial isolates (n = 10) were subjected to identification by the following criteria: Gram-staining, motility, oxidase, catalase, and API-20E test strips (BioMerieux, Inc., Hazelwood, MO, USA) following the manufacturer’s instructions (for Gram-negative isolates). The API strips were examined after 36 h of incubation at 28 °C, and positive and negative reactions in the different wells were noted, resulting in a seven-digit code. The API-20E profile code obtained for all isolates was interpreted according to the API numerical identification database (APIweb™). Subsequently, isolates to be archived were grown in Trypticase Soy Broth (TSB; Becton Dickinson, Sparks, MD, USA) for 24 h at 28 °C and were cryogenically preserved at −80 °C as standard 20% glycerol stocks.

### 2.4. DNA Extraction, PCR, and Sequencing

Genomic DNA was extracted from pure cultures of all the isolates using Qiagen Blood & Tissue DNeasy kit (Qiagen, Valencia, CA, USA) following the manufacturer’s guidelines. DNA was eluted with 100 μL elution buffer, quantified with a Thermo Scientific NanoDrop One^C^ Microvolume UV-Vis Spectrophotometer (Thermo Fisher Scientific Inc., Waltham, MA, USA), and stored at −20 °C for downstream nucleic acid analysis. A specific polymerase chain reaction (PCR) assay based on the *hug*A gene encoding the heme iron utilization system of *P. shigelloides* was used for molecular confirmatory identification of the isolates [25,26]. Primers described by Herrera et al. [26] targeting *hug*A were used to amplify a 435 bp fragment. The forward primer (*hug*A-F); 5′-GCG AGC GGG AAG GGA AGA ACC-3′, and the reverse primer (*hug*A-R); 5′-GTC GCC CCA AAC GCT AAC TCA TCA-3′. Genomic DNA of a previously characterized *P. shigelloides* isolate from Florida largemouth bass (*Micropterus salmoides*) was provided by the USDA-ARS Aquatic Animal Health Research Unit, Auburn, AL, and used as a reference (positive control). The PCR conditions in a final reaction volume of 50 μL followed those described by Herrera et al. [26]. ChemiDoc MP imager (Bio-Rad Laboratories, Inc., Hercules, CA, USA) was used to view the PCR products after 1% (*w*/*v*) agarose gel electrophoresis and ethidium bromide staining.

For sequencing, another PCR reaction to amplify the 16S rRNA gene of five representative isolates was performed as previously described [27] using the 16S universal Eubacterial primer set 27 F (5′-AGRGTTTGATCMTGGCTCAG-3′) and 519R (5′-GWATTACCGCGGCKGCTG-3′) targeting the hypervariable V1–V3 region. The PCR reactions (25 µL total volume) were carried out using 12.5 µL of EconoTaq PLUS GREEN 2X Master Mix (Lucigen, Middleton, WI, USA), 0.25 µL of each forward and reverse primer (final concentration 10 µM), 1 µL of template genomic DNA (30 ng/µL), and 11 µL of nuclease-free molecular grade water. The amplification was conducted on a T100™ Thermal Cycler (Bio-Rad Laboratories Inc., Hercules, CA, USA). PCR conditions were as follows: initial denaturation at 95 °C for 5 min, followed by 40 cycles of denaturation at 95 °C for 30 s, annealing at 55 °C for 30 s, extension at 72 °C for 45 s, a final extension at 72 °C for 5 min, and then holding at 4 °C. Aliquots from each PCR amplification reaction were verified by electrophoresis on a 1% (*w*/*v*) agarose gel stained with ethidium bromide and run at 100 V for 45 min. Bands were visualized using the ChemiDoc MP imager to confirm the presence and purity of single, specific bands. The PCR products were then purified using the QIAquick PCR purification kit (Qiagen, Valencia, CA, USA) according to the manufacturer’s instructions. Purified amplicons were subsequently submitted for Sanger sequencing at a commercial service (Source BioScience, Nottingham, UK), and sequence similarity was analyzed using BLASTn against the National Center for Biotechnology Information (NCBI) GenBank database.

### 2.5. Experimental Pathogenicity Challenges

Clinically healthy channel catfish (CC, n = 130, weighing 54.6 ± 3.8 g, mean ± SD) and hybrid catfish (HC, n = 130, weighing 45.1 ± 2.1 g, mean ± SD) juveniles were challenged by immersion or intraperitoneal injection (IP) using a representative *P. shigelloides* isolate (HM01-TX23). Briefly, prior to the challenge, ten fish of each stock were randomly screened microbiologically to ensure freedom from *P. shigelloides* infection. Then, ten catfish of each species were stocked into each 60 L glass aquarium and acclimated for one week before experimental infection. The challenge aquaria were supplied with flow-through de-chlorinated heated city water at an approximately 1.0 L/min flow rate and continuously aerated via air stones. Water quality parameters during the challenge were measured daily using an YSI ProODO meter (Yellow Springs Instrument, Inc., Yellow Springs, OH, USA) and were as follows: DO 6.93 ± 0.52 mg/L, water temperature 29.6 ± 0.6 °C, ammonia concentration 0.51 ± 0.08 mg/L, and pH 7.5 ± 0.4. A controlled photoperiod of 12:12 h L/D was used.

After acclimation, fish were divided into eight treatment groups: (I) CC *P. shigelloides* immersion; (II) CC *P. shigelloides* IP injection; (III) CC control (Mock-challenged) immersion; (IV) CC control (Mock-challenged) IP injection; (V) HC *P. shigelloides* immersion; (VI) HC *P. shigelloides* IP injection; (VII) HC control (Mock-challenged) immersion; and (VIII) HC control (Mock-challenged) IP injection. Each group consisted of 3 replicate tanks with 10 fish per tank. The tanks were randomly assigned to each treatment group. The challenge procedures followed our previously described protocols [28]. Briefly, for immersion exposure, the water flow was turned off in the tanks for a 1 h immersion challenge. A total of 100 mL of *P. shigelloides* overnight culture in TSB with a bacterial concentration of 2.5 × 10^9^ colony-forming units per milliliter (CFU/mL) was added into each immersion challenge tank containing 10 L of water, resulting in a final infectious dose of 2.5 × 10^7^ CFU/mL. At the end of the immersion exposure, continuous water flow was resumed for the duration of the challenge experiment. For IP injection groups, *P. shigelloides* culture in TSB was centrifuged at 2500 rpm for 10 min. Then, the cell pellet was suspended in sterile phosphate-buffered saline (PBS) to a concentration equivalent to McFarland No.2 turbidity standard (~6.0 × 10^8^ CFU/mL). Prior to injection, fish were anesthetized in a 20 L bucket filled with tank water containing 100 mg/L of tricaine methanesulfonate (MS-222) buffered with sodium bicarbonate to pH 7.0. Anesthetized fish were IP injected with 0.1 mL of *P. shigelloides* cell suspension. Mock-challenged control fish were exposed to the same procedures as challenged fish, but were immersed in sterile TSB medium or received 0.1 mL of sterile PBS intraperitoneally. To enumerate the average CFU/mL of *P. shigelloides* used for the challenge, a standard plate count in triplicate was performed. Fish were monitored daily, and mortality was recorded. Freshly dead fish were tested bacteriologically to confirm *P. shigelloides* as the cause of death. The challenge experiment was terminated ten days post-infection. A log-rank (Mantel-Cox) test was used to perform Kaplan–Meier survival analysis in GraphPad Prism (Version 10.6.1, GraphPad Software Inc., La Jolla, CA, USA) for comparing survival curves after the challenge.

### 2.6. Antimicrobial Susceptibility Testing

The Kirby–Bauer disc diffusion assay was used to profile the antimicrobial susceptibility/resistance patterns of ten isolates against the 3 antimicrobial agents approved by the U.S. Food and Drug Administration (FDA) for use in aquaculture: oxytetracycline 30 µg, florfenicol 30 µg, and romet-30 (a blend of ormetoprim and sulfadimethoxine [29]). All antimicrobials were obtained as commercial Sensi-Disc/50 disc cartridges or vials; oxytetracycline from BD (Becton Dickinson, Franklin Lakes, NJ, USA), florfenicol from Merck (Merck Animal Health, Inc., Summit, NJ, USA), and romet from AquaTactics (AquaTactics, Bimeda, Kirkland, WA, USA). The *Escherichia coli* ATCC 25922 strain was used as a quality control organism. Bacterial inocula at a concentration of 2.0 × 10^8^ CFU/mL (McFarland No. 0.5 turbidity standard) were prepared and streaked on Mueller–Hinton agar (BD, Franklin Lakes, NJ, USA) plates, with 3 replicates (plates) per isolate as previously described [30], following the manual on antimicrobial susceptibility testing procedures. After incubation at 28 °C for 48 h, the diameters in millimeters (mm) of the inhibition zones were measured. In accordance with the manufacturer’s guidelines, phenotypes were interpreted (to the nearest millimeter) as susceptible, intermediate, or resistant as follows: oxytetracycline > 19, 15−18, <14; florfenicol > 19, 15−18, <14; romet > 19, 14−18, <13 [31].

### 2.7. Histological Examination

Samples from the gastrointestinal tract (stomach and proximal and distal intestine) of immersion-infected and control channel catfish were collected and fixed in 10% neutral-buffered formalin for histological examination. Formalin-fixed tissues were processed routinely and embedded in paraffin-wax, sectioned at 5 μm, and stained with hematoxylin and eosin (H&E). Full-thickness sections evaluated included the anterior, transitional and pyloric gastric regions (n = 1 section), the proximal intestine (n = 3), and the distal intestine (n = 2). The degree of lesion severity was subjectively scored as none, minimal (1), mild (2), moderate (3), and marked (4), considering previous recommendations [32,33].

## 3. Results

### 3.1. Clinical Signs and Gross Autopsy Findings of Naturally Infected Fish

The disease outbreak among hybrid catfish was observed in 22 out of 60 growout ponds during the summer of the 2023 production season. Affected ponds experienced sustained daily mortalities for approximately four weeks, with an estimated 50–80 fish per pond per day, corresponding to a cumulative loss of approximately 1500–2400 fish per pond. The affected ponds were stocked with larger fish, averaging 1.0 ± 0.3 kg, whereas the healthy ponds contained smaller fish, weighing between 0.4 and 0.6 kg. Based on farm-reported stocking densities (20,000–25,000 lb per acre), pond sizes (1–8 acres), and estimated mortality counts, the cumulative mortality in affected ponds was estimated to range from 6% to 18% of the stocked population, depending on pond size and biomass. Although these values are approximate (as recorded by farm personnel), they highlight the substantial economic impact of the outbreak under commercial production conditions. According to the farmer, the only noticeable behavioral signs prior to the onset of mortality were reduced feeding response, decreased feed intake, and problems with buoyancy. The peak mortality coincided with the highest water temperatures, ranging from 28.7 °C to 32.2 °C.

**Figure 1:**
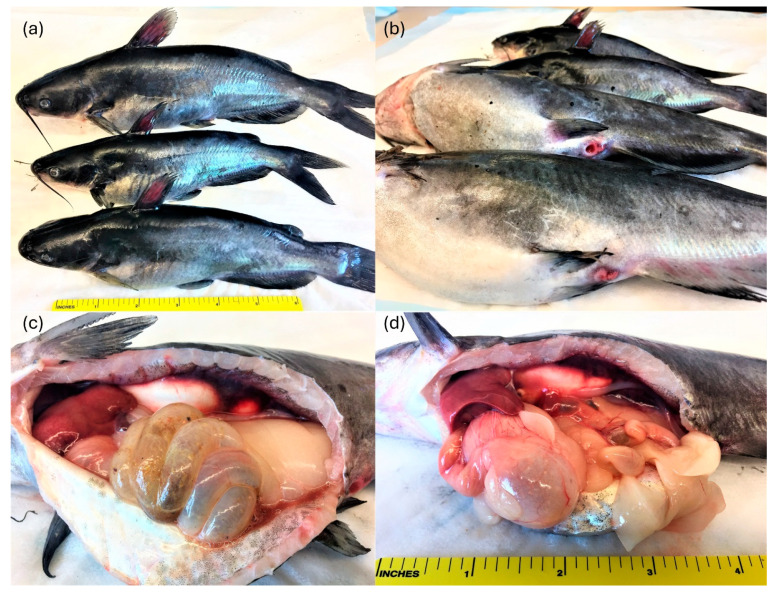
Hybrid catfish naturally infected by *Plesiomonas shigelloides*. Erythematous fins (**a**); swollen and erythematous vents and distended abdomens (**b**); and serosanguineous peritoneal effusion, mottled liver, abundant coelomic adipose tissue, and distended gastrointestinal tract (**c**,**d**).

Upon gross examination, the dead and moribund fish were in good nutritional body condition externally, with no evidence of emaciation or chronic malnutrition; however, they exhibited distended abdomens, erythematous dorsal fins, and swollen and erythematous vents (Figure 1), consistent with an acute disease process. Initial laboratory examination did not reveal any external pathogens or pathognomonic pathological changes. However, a comprehensive autopsy revealed visceral congestion, severe gastroenteritis, and serosanguineous effusion in the coelomic cavity (Figure 1). The fish had abundant coelomic adipose tissue, indicating that they were well fed. The livers were friable and mottled with petechiae; while splenomegaly and renomegaly were prominent. The most notable clinical finding was distension of the gastrointestinal (GI) tract, characterized by excessive contents and gas, resulting in abdominal distension. Microscopic examination of wet mounts showed only a mild gill fluke (*Dactylogyrus* spp.) infestation in the gills. No other parasites were detected in the skin and fin biopsies or in tissue squash preparations. The CCO cells did not exhibit CPE after 10 days of incubation at 25 °C or after the blind passage, indicating that no viruses were present in the culture.

### 3.2. Bacterial Identification, Molecular Confirmation, and Sequencing

**Figure 2:**
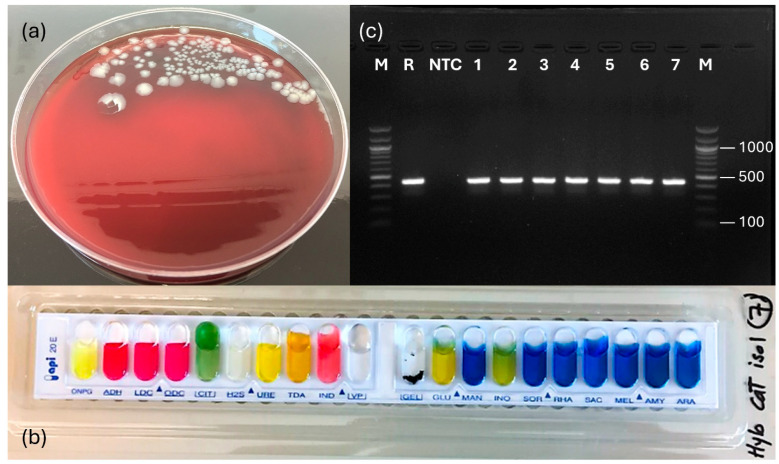
*Plesiomonas shigelloides* growth on blood agar plate showing a pure culture with a single colony morphotype (**a**); biochemical characterization of *P. shigelloides* isolates using commercial API strips yielding API-20E profile 7144204 (**b**); and agarose gel electrophoresis of polymerase chain reaction products (**c**) amplified from templates comprising DNA extracted from bacterial isolates recovered from naturally infected catfish (lane M: 100-base-pair [bp] ladder; lane R: reference *P. shigelloides*; lane NTC: no-template control; and lanes 1–7: genomic DNA from case isolates [435 bp]). Molecular weight markers (bp) are indicated on the sides of the gel.

After incubation at 28 °C for 48 h, a homogeneous growth of convex, cream-colored, opaque, and non-hemolytic colonies (a single colony type) appeared on all culture plates from the major internal organs (Figure 2a). The isolated bacteria were Gram-negative, rod-shaped, motile, oxidase-positive, and catalase-positive (Table 1). Initial presumptive biochemical characterization using commercial API strips yielded an API-20E profile 7144204 (Figure 2b), identifying *P. shigelloides* (99.9% ID accuracy). Table 1 summarizes the morphological and traditional biochemical attributes of the bacterial isolates. Molecular confirmation was achieved using a specific PCR assay targeting the *hug*A gene of *P. shigelloides*, which generated 435 bp amplicons (Figure 2c). To further explore the identity of the isolates, sequencing of the hypervariable V1–V3 region of the 16S rRNA gene confirmed the infection by *P. shigelloides*. BLASTn analysis of the sequences displayed 100% sequence similarity to the *P. shigelloides* strain MS-17-188 chromosome (accession no. CP027852.1).

### 3.3. In Vivo Pathogenicity Assays

**Figure 3:**
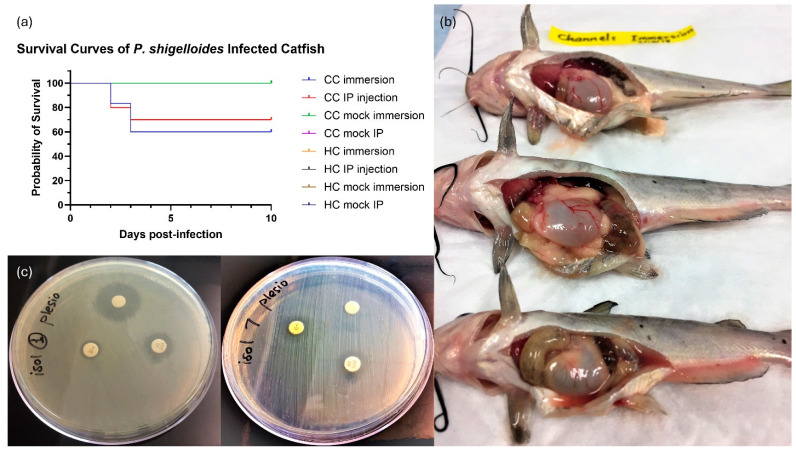
Survival curves of experimentally infected channel and hybrid catfish with *P. shigelloides* showing 60% survival in cc immersion and 70% survival in CC IP injection (**a**). All other treatments had 100% survival (curves are superimposed); experimentally infected channel catfish with *P. shigelloides* via immersion displaying bloody ascitic fluid in the coelomic cavity and distended gastrointestinal tract (**b**). Antimicrobial resistance of two *P. shigelloides* isolates showing reduced susceptibility to all FDA-approved antimicrobials (**c**): oxytetracycline (T30), florfenicol (FFC30), and Romet-30 (blank disc).

Experimental infection of channel catfish with *P. shigelloides* resulted in significant mortality following both immersion exposure (treatment I) and IP injection (treatment II). Cumulative mortalities were 40.0 ± 5.78% and 30.0 ± 5.78% (corresponding to 60% and 70% survival), respectively, whereas no mortalities (0%) were observed in the route-matched control groups (treatments III and IV) (Figure 3a). All mortalities in the challenged groups occurred within the first 3 days post-challenge, from day 2 to day 4. Dead fish exhibited gastroenteritis and multicavitary serosanguineous effusion (Figure 3b), similar to those observed on the naturally infected fish. Although the infected hybrid catfish in treatments V and VI showed sluggish movement and lethargy a few hours after infection, no mortalities were recorded. There were no mortalities in any of the control/Mock-challenged groups (treatments III, IV, VII, and VIII). Kaplan–Meier survival curves for all groups were compared using a log-rank (Mantel–Cox) test, which revealed a highly significant difference among treatments (χ^2^ = 73.94, df = 7, *p* < 0.0001). Isolation attempts from the internal organs of freshly dead fish recovered large numbers of bacteria consistent with *P. shigelloides*. No *P. shigelloides* could be isolated from the survivors at the termination of the trial (10 days post-infection).

### 3.4. Antimicrobial Susceptibility

Based on the diameter of the growth inhibition zones, all tested *P. shigelloides* isolates (n = 10) demonstrated reduced susceptibility to all the FDA-approved antimicrobials evaluated. The mean inhibition zone diameters (in mm ± SD) were relatively small across isolates for all three drugs tested (oxytetracycline: 7.0–11.4 ± 0.3 mm; florfenicol: 8.6–12.3 ± 0.2 mm; Romet: 6.0–12.1 ± 0.3 mm), suggesting reduced susceptibility compared with typical values reported for susceptible aquatic isolates (no reference *P. shigelloides* isolate with known susceptibility was available for formal statistical comparisons). Figure 3c shows the antimicrobial resistance profiles of two representative *P. shigelloides* isolates on Mueller–Hinton agar.

### 3.5. Histopathological Assessment

**Figure 4:**
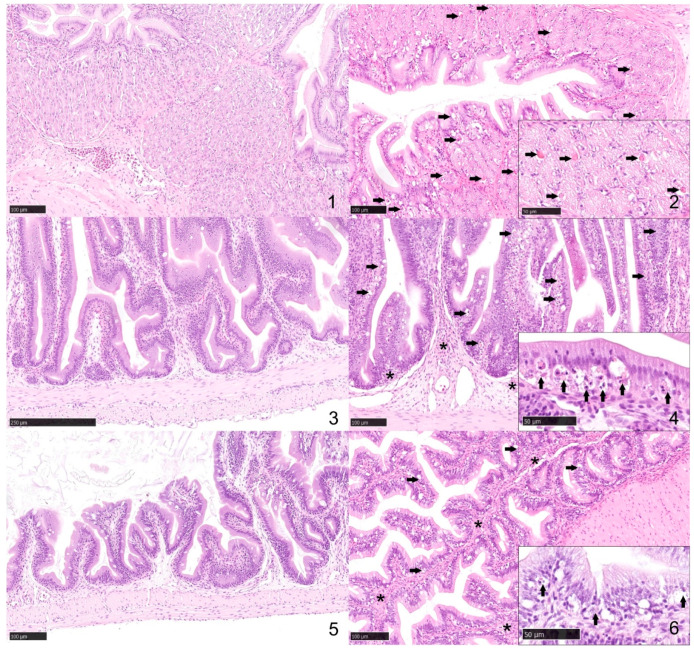
*Plesiomonas shigelloides* experimental infection in channel catfish (*Ictalurus punctatus*). Histological findings in the gastrointestinal tract of control (sections **1**,**3**,**5**) and immersion-infected catfish (sections **2**,**4**,**6**). **1.** Stomach. Unremarkable, normal gastric mucosa. Hematoxylin and eosin (H&E), 200×. **2.** Stomach. The glandular epithelium exhibits single cell necrosis/apoptosis (arrows). H&E, 200×. Inset: degenerating glandular epithelium (arrows). H&E, 400×. **3.** Proximal intestine. The normal lamina propria has a minimal number of lymphocytes and the epithelium is diffusely intact. H&E, 200×. **4.** Proximal intestine. The lamina propria is infiltrated by small numbers of lymphocytes and granulocytes (asterisks). The superficial epithelium is disrupted and there is frequent single cell necrosis/apoptosis (arrows) and leukocyte transepithelial migration. H&E, 200×. Inset: degenerating epithelium with inflammatory cells, cellular debris (arrows) and edema. H&E, 400×. **5.** Distal intestine. The normal lamina propria has a minimal number of lymphocytes and the epithelium is diffusely intact. H&E, 200×. **6.** Distal intestine. Note similar changes to those in the proximal intestine. Fewer inflammatory cells (asterisks) infiltrate the lamina propria. The superficial epithelium is disrupted and there is frequent single-cell necrosis/apoptosis (arrows) and leukocyte transmigration. H&E, 200×. Inset: degenerating epithelium with inflammatory cells, cellular debris (arrows) and edema. H&E, 400×.

Lesion patterns noted were circulatory disturbances, degenerative changes, adaptive changes, and inflammatory changes (modified from Bernet et al. and Rey et al. [32,33]). In the sections examined from the control catfish, there was evidence of none to minimal inflammatory or degenerative changes. The stomach sections showed normal gastric mucosa. The lamina propria had a minimal number of lymphocytes and fewer plasma cells (<100 lymphocytes in 0.2 mm^2^). None or very rare granulocytes were noted in the lamina propria. The lamina propria of the proximal and distal intestine had a minimal number of lymphocytes, few plasma cells, and very rare heterophils (Figure 4). On the contrary, in the stomach sections from infected catfish, there was mild, multifocal, subacute pleocellular lymphocytic gastritis with moderate glandular granular epithelial cell necrosis/apoptosis. The lamina propria was infiltrated by a small number of lymphocytes and fewer plasma cells. Rare granulocytes with variably depleted cytoplasmic granules were noted. Multifocally, the superficial epithelium, more prominently along the basal region, appeared vacuolated. Glandular granular cells appeared consistently slightly depleted (increased clear space), and there was moderate single-cell necrosis/apoptosis. Small numbers of bacteria were noted in the lumen. The proximal and distal intestine sections had moderate, multifocal, subacute pleocellular lymphocytic and granulocytic enteritis with epithelial degeneration and necrosis/apoptosis. In the lamina propria, and to a lesser extent in the submucosa, there was infiltration by moderate numbers of lymphocytes along with fewer plasma cells and granulocytes often with depletion of cytoplasmic granules. Multifocally, the superficial epithelium, more prominently along the basal region, appeared vacuolated. Single cell necrosis/apoptosis of primarily epithelial cells was common throughout multiple affected areas. Transepithelial leukocyte migration was also common. The vasculature was hyperemic/congested and lined by hypertrophic endothelium, and there was mild edema. Multifocally, the serosal mesothelium appeared plump, and there were rare lymphocytes. Small numbers of bacteria were noted in the lumen.

## 4. Discussion

In commercial aquaculture, the incidence of infectious diseases has increased as culture systems expand and intensify to meet the growing demand for seafood [34,35]. Over the past decade, emerging pathogens have posed significant challenges for the U.S. and global aquaculture sectors [6,36,37,38]. This study documents the first confirmed case of *P. shigelloides* infection associated with clinical disease and mortality in cultured hybrid catfish on a commercial South Texas farm. Our findings, encompassing clinical signs, pathology, microbiological identification, molecular confirmation, fulfillment of Koch’s postulates, and antimicrobial susceptibility testing, collectively highlight the growing concern of *P. shigelloides* as an emerging catfish pathogen in the U.S. catfish industry.

The recovery of a homogenous growth of *P. shigelloides* from the major internal organs of moribund fish, coupled with the absence of other significant pathogens (viral or parasitic), strongly implicates this bacterium as the primary cause of mortality. Conditions that triggered the onset of this disease outbreak remain largely unknown. *P. shigelloides* is commonly found in freshwater fish and aquatic environments as a commensal microorganism. In the southeastern U.S., it is the predominant bacterial species isolated from the gut microbiota of catfish, as well as from catfish pond sediment [13,14]. However, its role as a primary pathogen in aquaculture has been increasingly recognized in recent years, demonstrating a broad host tropism. Previous disease reports involving *P. shigelloides* have included cases in catfish [39], tilapia [40,41], carps [42,43], rainbow trout [12], eel [44], snakehead [45], and ornamental species [10,46], where the organism was linked to septicemia, gastroenteritis, abdominal distension, and hemorrhagic inflammatory lesions. Likewise, during the summer of 2017 (May–July), we characterized a similar acute gastroenteritis outbreak caused by bacterial co-infection involving *P. shigelloides* and *Lactococcus garvieae* in hybrid catfish within a floating in-pond raceway system in a research fish population at Auburn University, AL [47]. The present study expands this knowledge by associating *P. shigelloides* with mortality in intensively stocked hybrid catfish ponds, suggesting that the organism is capable of causing substantial economic losses in commercial catfish aquaculture during the warm summer months when environmental or host-related stressors compromise fish health.

A key pathological feature observed during this outbreak was the presence of severe enteropathogenicity (gastroenteritis and serosanguineous peritoneal effusion), which aligned closely with the classical enteric disease manifestations previously reported for *P. shigelloides* in fish and humans. *P. shigelloides* is well known in human medicine as an etiological agent of acute and chronic gastroenteritis, with virulence determinants that include cytotoxins, enterotoxins, hemolysins, multiple secretion system effectors, invasion factors, and biofilm formation [10,48,49,50,51]. Additionally, *P. shigelloides* has been mainly associated with outbreaks of gastrointestinal diseases due to the consumption of fish [52]. The pronounced enteric inflammation, glandular necrosis, and epithelial necrosis/apoptosis observed histologically in the experimentally challenged channel catfish strongly suggest that *P. shigelloides* has a primary gastrointestinal tropism as previously reported by Wang et al. [37] in channel catfish. The severe accumulation of gas and ingesta within the GI tract likely contributed to buoyancy dysfunction, impaired physiological performance, and eventual mortality, especially under high-temperature and high-density production conditions in commercial aquaculture ponds.

The pathogenicity to channel catfish observed in our experimental challenges further supports the conclusion that *P. shigelloides* isolates recovered from the field mortalities were pathogenic, fulfilling Koch’s postulates. However, the pathogenesis of *Plesiomonas*-associated gastroenteritis still remains unknown. While intraperitoneal injection is often used to bypass mucosal defenses, the significant mortality following immersion challenge suggests that *P. shigelloides* is capable of infecting catfish through natural routes (e.g., gills, skin, or gut). The *in vivo* trials demonstrated a rapid onset of mortality in channel catfish, within 3 days post-infection. Importantly, the experimentally infected channel catfish reproduced the same clinical signs of gastroenteritis and serosanguineous peritoneal effusion as seen in the natural outbreak (Figure 1 and Figure 3), whereas hybrid catfish did not exhibit any noticeable clinical signs or mortality. A recent study by Abdelhamed et al. 2024 [53] demonstrated that *P. shigelloides* was unable to establish infection in channel catfish and was cleared without causing mortality. This also suggests that *P. shigelloides* may include both pathogenic and non-pathogenic strains. Additionally, pathogenicity may be influenced by coinfection [47] or host-related factors, such as disruption of epithelial barriers or a compromised immune system, which could allow otherwise non-pathogenic strains to cause disease under certain conditions. Factors that may contribute to the differences in mortality between channel catfish and hybrid catfish include host species-specific differences in susceptibility, immune responses, and interactions between the gut microbiome and the pathogen. Hybrid catfish are generally considered more disease-resistant than pure channel catfish, which may partially explain their survival post-infection in the lab [6,54,55]. Hybrids are also more resistant to stressors than channel catfish [56], which are considered a prerequisite to bacterial infections, especially those caused by opportunistic pathogens. Environmental or physiological cofactors in the production setting, including high water temperatures (>28 °C), high biomass loading, fish size, intensive feeding, and abundant visceral fat reserves, could have synergistically exacerbated the natural disease progression in hybrid catfish, whereas controlled laboratory conditions may have limited these variables. Taken together, our field observations and laboratory challenge results suggest several testable hypotheses regarding potential triggers of *P. shigelloides*–associated disease in hybrid catfish. While controlled laboratory conditions were sufficient to reproduce disease in channel catfish, the absence of mortality in experimentally challenged hybrid catfish indicates that additional host–pathogen–environment interactions present under commercial production conditions may be required to precipitate disease. Future studies explicitly manipulating these variables will be necessary to define their relative contributions to outbreak initiation and severity. Such findings emphasize the importance of further comparative pathogenicity and immunological studies between channel and hybrid catfish strains to better understand differential host resilience.

Of considerable concern is the reduced susceptibility phenotype demonstrated across all tested *P. shigelloides* isolates. Resistance to oxytetracycline, florfenicol, and Romet-30, which represent the only FDA-approved antimicrobials for use in U.S. food-fish aquaculture, poses a significant challenge for disease management. The resistance patterns observed in this study are consistent with previous reports of antimicrobial resistance among *P. shigelloides* strains isolated from diseased catfish [39,53], likely driven by environmental antibiotic exposure, selective pressure from medicated feeds, and horizontal gene transfer in aquatic microbial communities. The Aquatic Diagnostic Laboratory at the College of Veterinary Medicine, Mississippi State University, recovered a multidrug-resistant *P. shigelloides* strain MS-17-188 from a diseased catfish in 2017, which was resistant to gentamicin, tetracycline, oxytetracycline, doxycycline, sulfamethoxazole-trimethoprim, florfenicol, erythromycin, chloramphenicol, streptomycin, azithromycin, penicillin, novobiocin, and spectinomycin. The complete genome sequence of *P. shigelloides* MS-17-188 included a single contig chromosome and three circular plasmids, and one of the three plasmids (pPS-MS-17188-3) was found to carry several antibiotic resistance genes [39,53]. Moreover, since *P. shigelloides* is a recognized foodborne pathogen in humans, and the isolated bacteria demonstrated reduced susceptibility to antimicrobials, this raises potential public health considerations. Although this study did not assess human health risk or resistance determinants associated with the clinical isolates, our findings highlight the importance of antimicrobial stewardship in aquaculture, adherence to drug withdrawal periods, and proper handling and thorough cooking of catfish products to minimize potential food safety risks.

Given that the *P. shigelloides* isolates in this study were resistant to all FDA-approved antimicrobials available, no effective chemotherapeutic options were available for treatment. As a management intervention, fish were held off feed for one week, after which the fish responded positively and the outbreak resolved spontaneously. This outcome is consistent with the generally self-limiting nature of *P. shigelloides* gastroenteritis reported in 85% of human cases [57,58] and suggests that reducing gut content and metabolic demand may help mitigate disease severity during outbreaks. With treatment options currently limited and ineffective, this highlights the urgent need for alternative prevention and mitigation strategies, including vaccination, feed-based immunostimulants, probiotics, improved biosecurity, reduced stocking densities, and water-quality-driven management interventions. Additionally, comparative genomic analysis of many *P. shigelloides* isolates is warranted to elucidate their diversity, resistance gene profiles, virulence determinants, and epidemiological patterns.

## 5. Conclusions

In high-density commercial aquaculture settings, *P. shigelloides* could become recurrent, and potentially difficult to manage using currently approved antimicrobials. Collectively, the findings we presented demonstrate that *P. shigelloides* should be recognized as an emerging enteric pathogen of concern in catfish aquaculture, particularly under intensive production regimes and suboptimal culture conditions that favor pathogen proliferation and host vulnerability. Preventive management, early diagnostic screening, and proactive monitoring should be prioritized to minimize the risk of future economic losses. Continued research into host–pathogen-environment interactions and development of evidence-based control strategies will be critical to protecting fish from future *P. shigelloides* outbreaks within the rapidly expanding hybrid catfish aquaculture industry.

## Figures and Tables

**Table 1:** Biochemical properties of *Plesiomonas shigelloides* isolates from hybrid catfish. Reading of the API-20E strips and interpretation of results are in accordance with the manufacturer’s (BioMerieux) recommendations. Number of isolates tested n = 10.

Test	Biochemical Characteristics
Gram staining	−
Motility	+
Catalase test	+
Cell morphology	Rod-shaped
**API 20E:**	
ONPG test	+
Arginine dihydrolase	+
Lysine decarboxylase	+
Ornithine decarboxylase	+
Citrate utilization	−
H_2_S production	−
Urea hydrolysis	−
Tryptophan deaminase	−
Indole production	+
Voges–Proskauer (Acetoin production reaction)	−
Gelatin hydrolysis	−
Glucose fermentation/oxidation	+
Mannitol fermentation/oxidation	−
Inositol fermentation/oxidation	+
Sorbitol fermentation/oxidation	−
Rhamnose fermentation/oxidation	−
Sucrose fermentation/oxidation	−
Melibiose fermentation/oxidation	−
Amygdaline fermentation/oxidation	−
Arabinose fermentation/oxidation	−
Oxidase	+

+, 100% positive isolates; −, 100% negative isolates.

## Data Availability

The original contributions presented in this study are included in the article. Further inquiries can be directed to the corresponding author.

## Abbreviations

The following abbreviations are used in this manuscript:

PCR	Polymerase chain reaction
FDA	Food and Drug Administration
rRNA	Ribosomal RNA
U.S.	United States
USDA-NASS	United States Department of Agriculture-National Agricultural Statistics Service
FHDL	Fish Health & Disease Laboratory
DO	Dissolved oxygen
CCO	Channel catfish ovary
ATCC	American Type Culture Collection
USFWS	U.S. Fish and Wildlife Service
AFS-FHS	American Fisheries Society-Fish Health Section
CPE	Cytopathic effect
TSB	Trypticase Soy Broth
NCBI	National Center for Biotechnology Information
CC	Channel catfish
HC	Hybrid catfish
IP	Intraperitoneal injection
CFU	Colony-forming units
PBS	Phosphate-buffered saline
MS-222	Tricaine methanesulfonate
H&E	Hematoxylin and eosin
